# The use of a new calcium mesoporous silica nanoparticle versus calcium and/or fluoride products in reducing the progression of dental erosion

**DOI:** 10.1590/1678-7757-2020-0131

**Published:** 2020-07-24

**Authors:** Fernanda Michel Tavares CANTO, Adílis Kalina ALEXANDRIA, Isabela B. dos Santos JUSTINO, Gustavo Miranda ROCHA, Lúcio Mendes CABRAL, Raphael da Silva FERREIRA, Matheus Melo PITHON, Lucianne Cople MAIA

**Affiliations:** 1 Universidade Federal do Rio de Janeiro Departamento de Odontopediatria e Ortodontia Rio de JaneiroRJ Brasil Universidade Federal do Rio de Janeiro, Departamento de Odontopediatria e Ortodontia, Rio de Janeiro, RJ, Brasil.; 2 Universidade Federal do Rio de Janeiro Instituto de Biofísica Carlos Chagas Filho Departamento de Biofísica Rio de JaneiroRJ Brasil Universidade Federal do Rio de Janeiro, Instituto de Biofísica Carlos Chagas Filho, Departamento de Biofísica, Laboratório de Física Biológica, Rio de Janeiro, RJ, Brasil.; 3 Universidade Federal do Rio de Janeiro Faculdade de Farmácia Departamento de Medicamentos Rio de JaneiroRJ Brasil Universidade Federal do Rio de Janeiro, Faculdade de Farmácia, Departamento de Medicamentos, Rio de Janeiro, RJ, Brasil.; 4 Universidade Estadual do Sudoeste da Bahia Departamento de Ortodontia JequiéBA Brasil Universidade Estadual do Sudoeste da Bahia, Departamento de Ortodontia, Jequié/BA, Brasil.

**Keywords:** Tooth erosion, Mesoporous silica nanoparticles, Fluoride compounds, Tooth remineralization

## Abstract

**Objective:**

There is increasingly common the consumption more times a day of foods and acidic drinks in the diet of the population. The present study aimed to evaluate and compare the effects of a calcium mesoporous silica nanoparticle single application of other calcium and/or fluoride products in reducing the progression of dental erosion.

**Methodology:**

Half of the eroded area was covered of 60 blocks of enamel, after which the block was submitted to the following treatments: (Ca^2+^-MSN), casein phosphopeptide–amorphous calcium phosphate (CPP-ACP); CPP-ACP/F-(900 ppm F−); titanium tetrafluoride (TiF_4_ 1%) (positive control); sodium fluoride (NaF 1.36%) (positive control); and Milli-Q^®^ water (negative control) before being submitted to a second erosive challenge. A surface analysis was performed via a three-dimensional (3D) noncontact optical profilometry to assess the volumetric roughness (Sa) and tooth structure loss (TSL) and and through scanning electron microscopy (MEV). An analysis of variance (ANOVA) and Tukey’s test were performed.

**Results:**

Regarding Sa, all experimental groups exhibited less roughness than the control (p<0.05). The TSL analysis revealed that the Ca^2+^-MSN and NaF groups were similar (p>0.05) and more effective in minimizing tooth loss compared with the other groups (p<0.05).

**Conclusions:**

The Ca^2+^-MSN and NaF treatments were superior compared with the others and the negative control.

## Introduction

Teeth can be exposed to acidic compounds during normal daily activities, especially during the consumption of soft drinks and juices, while taking medications with an acidic composition, or after being subjected to intrinsic acids such as esophageal reflux, these compounds can cause an irreversible and progressive loss of tooth enamel.^1,2^ As a result, many clinical consequences arise, including tooth sensitivity or the loss of dental structure around the restorations, which can lead to a gap that may eventually progress to dentin exposure.^3^

For these reasons, products exist to help minimize the evolution of existing erosion lesions. Fluorides are the adjuncts most commonly used to prevent further enamel structure loss, as they form a protective layer of calcium fluoride (CaF_2_) over the enamel surface.^3^This CaF_2_ layer serves as a mineral reservoir on the enamel surface and, in cases of demineralization, it is the first to be dissolved.^4^ Other fluoride products such as titanium tetrafluoride (TiF_4_) act by forming a titanium oxide film that prevents erosion.^5,6^ In addition, researchers have explored the use of calcium-based products such as CPP-casein phosphopeptide, and ACP- amorphous calcium phosphate paste; and CPP-ACP/F^−^ in the remineralization of dental enamel. CPP provides calcium and phosphate at the enamel surface and which acts in the demineralization-remineralization process.^7,8^ Amorphous calcium phosphate, when placed in an acidified pH solution, separates from the CPP, leaving the phosphate and calcium ions to interact with the dental enamel, helping to prevent mineral loss.^9^

Mesoporous silica has great research relevance in the area of health, and with wide application in the field of biomaterials, since it has a great capacity to incorporate molecules within its numerous pores present in its structure and release them in a sustained manner.^10^ Positive results have previously been found following use of a novel calcium mesoporous silica nanoparticle (Ca^2^-MSN) to prevent dental caries (unpublished data). In this formulation, silica is present as a mesoporous nanocomposite with high adsorption and is thus able to incorporate gradual-release compounds. Mesoporous silica is a nanocomposite that is capable of incorporating several compounds for its gradual release. In the case of our study, calcium was incorporated as the objective was to release calcium gradually. However, other compounds such as NaF, TiF_4_ (unpublished data), substances for medical use are also used.^11^

In addition, the nanocomposite silica has a high surface area since it has a large amount of hexagonal-shaped pores, which increases the loading of substances inside;^11^ and adequate thermal stability, that is, mesoporous silica can resist changes in temperature, without however changing its initial composition.^12^ Its application has been studied as a means to carry compounds and promote their slow release on an applied surface,^13^ which could interfere with mineral loss kinetics.

Therefore, based on the prior established benefits of the novel calcium mesoporous silica material in caries prevention and treatment, since calcium can minimize mineral loss whether in the process of caries or tooth erosion, so, the present study aimed to evaluate and compare the effects of a single application of this compound to those of other calcium and/or fluoride products, specifically in regard to reducing the progression of dental erosion.

## Methodology

This *in vitro* study evaluated the effects of a single application of Ca^2^-MSN in reducing the progression of dental erosion and compared the findings to other calcium and/or fluoride products. The choice of 10 blocks per group was made after performing a sample calculation to detect a significant 50% difference in mean mineral loss in each treatment group as compared with the negative control group while considering a statistical power of 80%, a unilateral test, and a significance level of 5%.^14^ The BioEstat 5.3 statistical analysis software program (Institute of Sustainable Development Mamirauá, Tefé, Amazonas, Brazil) was used to successfully detect a significant 50% difference in mean mineral loss in each treatment group in comparison with in the control group, considering a statistical power of 80%, a unilateral test, and a significance level of 5%. The blocks were exposed to an erosive challenge via immersion in a low-pH solution followed by immersion in another solution with neutral pH and a single application of the following products: (1) Ca^2^-MSN solution; (2) CPP-ACP slurry (CG America, Alsip, IL, USA); (3) CPP-ACP/F^−^ slurry (CPP-ACP + 900-ppm concentration of F^−^ from GC Corp., Tokyo, Japan); (4) TiF_4_ solution (1%); or (5) sodium fluoride solution (NaF 1.36%); or (6) Milli-Q^®^ water (Millipore Corp., Burlington, MA, USA) as a negative control. Subsequently, the blocks were one again exposed to *in vitro* erosive challenges and the topography of each block was analyzed via three-dimensional (3D) noncontact optical profilometry and scanning electronic microscopy (SEM).

### Sample preparation

**Figure 1:**
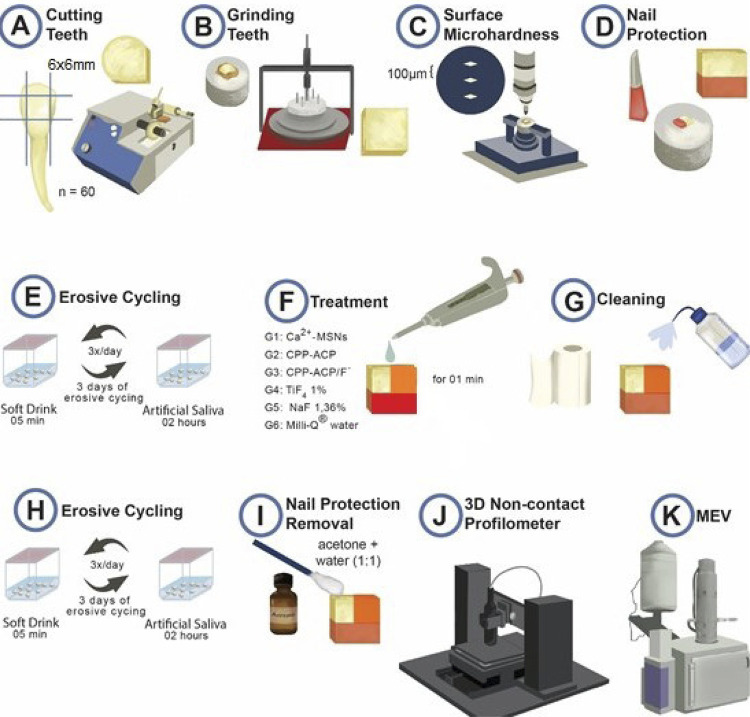
Schematic drawing of methodological steps. *erosive challenge before the treatments adapted from Canto, et al.27 (2020).

Sixty enamel blocks measuring 6×6×2 mm were cut using an Isomet low-speed saw cutting machine (Buehler Ltd., Lake Bluff, Illinois, United States) with two diamond discs (Extec Corp., Enfield, Connecticut, United States) and polished using water-cooled silicon carbide paper 600, 800 and 1,200 (Extec Corp., Enfield, Connecticut, United States) (Figure 1). To select the blocks, microhardness tests were performed following the methodology proposed by^15^ (Figures 1A, 1B, and 1C), a using a microhardness machine (Micromet 5104; Buehler, Mitutoyo Corporation, Tokyo, Japan) and the blocks that obtained the same average 342,90 (Kg/mm^2^) ± 10% were selected for the experiment.

### Treatments and erosive challenge

The enamel blocks (n=60) were randomly assigned into six groups (n=10 each) according to the proposed surface treatment. Half of each block was covered with an acid-resistant varnish (untreated area) (Figure 1D) and the other half (i.e., the eroded area) was submitted to the first erosive challenge for three days, three times daily, for five minutes (Figure 1E) in a low-pH solution (2.58; Sprite Zero™, Coca-Cola Co., Atlanta, GA, USA), according to the method proposed by.^16^ The blocks were then immersed in artificial saliva (composed of 1.5 mmol/L of calcium, 0.9 mmol/L of phosphate, 0.15 M of potassium chloride, Tris buffer, and 0.05 μg of fluoride/mL)^17^for two hours. Upon completion of the first erosive challenge, half of the eroded area was subsequently covered with the acid-resistant varnish and received a single application of a certain treatment from among the following (n=10 per group): experimental Ca^2^-MSN solution; CPP-ACP slurry; CPP-ACP/F^−^ slurry (CPP-ACP + 900-ppm concentration of F^−^); TiF_4_ solution (1%; positive control); and NaF solution (1.36%; positive control). The negative control samples (n=10) received a single application of Milli-Q^®^ water (Figure 1F).

The CPP-ACP (Pure Water, Glycerol, CPP-ACP, D-Sorbitol, Silicon Dioxide, CMC-Na, Propylene glycol, Titanium dioxide, Xylitol, Phosphoric acid, Guar gum, Zinc Oxide, Sodium Saccharin, Ethyl p-hydroxybenzoate, Propyl p-hydroxybenzoate) and CPP-ACP/F^−^ (Pure Water, Glycerol, CPP-ACP, D-Sorbitol, Silicon Dioxide, CMC-Na, Propylene glycol, Titanium dioxide, Xylitol, Phosphoric acid, Sodium fluoride, Guar gum, Zinc Oxide, Sodium Saccharin, Ethyl p-hydroxybenzoate, Propyl p-hydroxybenzoate) pastes were established as slurry with a 1:3 composition of one part toothpaste and three parts distilled and deionized water (i.e., Milli-Q^®^).^18^ The solutions of novel Ca^2^-MSN (1g of mesopourous silica doped with calcium powder to 100 ml of Milliq^®^ water), TiF_4_ (1,0% of powder concentration to 100ml of Milliq^®^ water) , and NaF (1.36% of powder concentration to 100ml of Milliq^®^ water) were developed in the Laboratory of Industrial Pharmaceutical Technology, Federal University of Rio de Janeiro in Rio de Janeiro, Brazil.

The new Mesoporous Silica was developed in the Laboratory of Industrial Pharmaceutical Technology, Federal University of Rio de Janeiro in Rio de Janeiro, Brazil. and after its synthesis, calcium particles were incorporated. It consisted, initially, in the development of an aqueous solution of NaOH, using H2O Milliq^®^, submitted to magnetic stirring. Then, cetyltrimethylammonium bromide (CTAB) was added, followed by tetraethoxysilane (TEOS), to obtain MNS. Soon after, Ca (NO_3_)_2_ was added in order to form a precipitate.

A one-minute, single application of each product on the relevant enamel-block surfaces was conducted with the aid of a 100-μL pipette. These surfaces were then washed and dried using absorbent paper (Figure 1G). The enamel blocks of the negative control group received distilled and deionized water (i.e., Milli-Q^®^) for the same period. After this treatment, all blocks were submitted to a second erosion challenge, as previously described (Figure 1H).

### Analysis in noncontact three-dimensional profilometry

After chemically removing the nail varnish protective covering with acetone (Figure 1I), block topography was evaluated (Figure 1). The topographical measurements of the various block areas were recorded using a noncontact 3D profilometer (Nanovea PS50 Optical; Nanovea Inc., Irvine, CA, USA), based on a methodology previously described.^19^ Measurements of volumetric roughness (Sa) were taken for both eroded (Sa_initial_) and treated areas (Sa_final_), with an area of 200×200 µm. The difference between the initial and final roughness findings was determined by the following: R = Sa_final_ − Sa_initial)_ For tooth structure loss (TSL) analyses, differences between the heights of the eroded and treated areas were analyzed. A qualitative analysis was also performed via 3D image profiling of each surface, considering a color scale varying from red (high peaks with low structure loss) to dark blue (deeper valleys with greater structure loss). All analyses were performed by a blinded examiner who only had access to a random number of the previously demarcated specimens (Figure 1J).

### Analysis using scanning electron microscopy

A SEM system was used to qualitatively analyze the enamel surface and the differences between eroded and treated areas as well as the nature of the step formed between them. All areas were covered with a thin layer of gold and analyzed by SEM (Fei Quanta 250; Czech Republic). The SEM operated between 15 kV and 20 kV, with an increased magnification of 500× applied at the interface areas. Photomicrographs were obtained to show the microstructural characteristics of the untreated, eroded, and treated areas (Figure 1K).

### Statistical analysis

The Shapiro–Wilk test was applied to evaluate the normality of Sa and TSL data through the Statistical Package for the Social Sciences software version 22.0 (IBM Corp., Armonk, NY, USA). Then, a one-way analysis of variance and Tukey’s test were used to evaluate Sa and TSL measurements between the eroded and treated areas. The level of significance was 95% (p<0.05). The SEM findings were used to explain surface alterations on the areas and differences between untreated, eroded, and treated areas.

## Results

The negative control blocks presented higher mean values of Sa in comparison with the difference between the eroded and treated areas of the experimental blocks (p<0.05). Furthermore, the studied products were able to reduce the mean difference of Sa between the eroded and treated areas as compared with the positive control (p<0.05), without statistical differences (p>0.05) (Table 1).

**Table 1:** Mean ± standard deviation of differences in volumetric roughness (S) and TSL (trated-eroded área) of enamel after application for each product and erosive challenge

	S (Sa treated- Sa eroded)	TSL ( treated area-eroded area)
	Mean (SD)	Mean (SD)
Ca2+-MSNs	-0,17(±0,35)a	11,91 (±2,04)a
CPP-ACP	- 0,11(±1,55)a	17,99(±5,04)b
CPP-ACP/F	-0,83(±2,45)a	19,07 (±13,79)b
TiF4	- 0,21(1,07)a	12,79(±5,00)b
NaF	-0,22(±0,46)a	9,83(±4,20)a
Water	-1,51(±1,90)b	33,39(±13,35)b

Based on the findings of TSL difference between the eroded and treated areas, the Ca^2^-MSN and NaF treatments were found to be similar in terms of effect (p>0.05) and thus more successfully reduced erosion progression than did CPP-ACP, CPP-ACP/F^−^, or TiF_4_. These latter treatments yielded results similar to those of the negative control (p<0.05) (Table 1).

**Figure 2:**
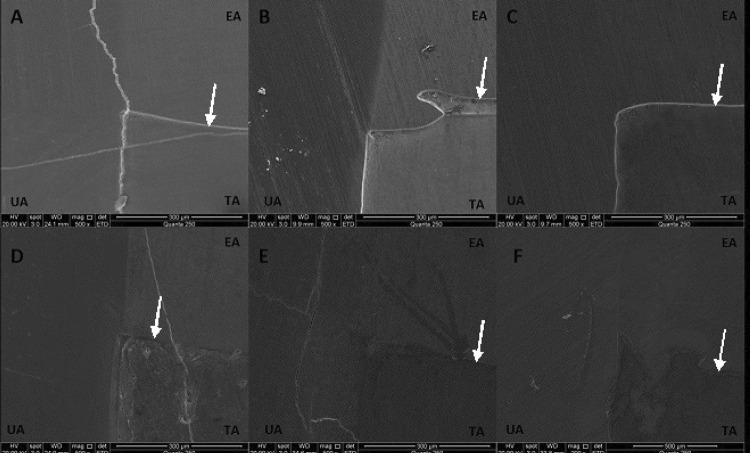
Surface SEM images of enamel of untreated area, eroded area and after treatment in 500X (image of the interface: untreated, eroded and treated areas). (A) Ca2+-MSN, (B) CPP-ACP, (C) CPP-ACP/F- , (D) TiF4, (E) NaF, (F) Negative Control (Water). In the images: UA: untreated area; EA: eroded area; TA: treated area. The white arrows represent the border area between EA and TA, where irregular enamel loss and the formation of steps are observed

**Figure 3:**
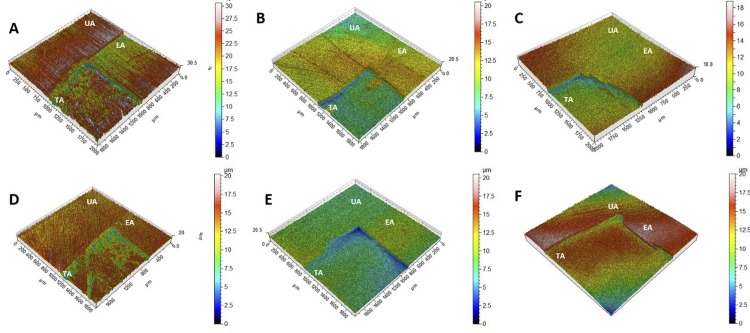
3D profilometry scheme representation of samples before and after erosive challenge. (A) Ca2+-MSN, (B) CPP-ACP, (C) CPP-ACP/F-, (D) TiF4 Control, (E) NaF Control, (F) Negative Control (Water). UA: untreated área;EA: eroded área;TA:treated área

When the SEM images (Figure 2) were reviewed, the groups that best inhibited the progression of enamel loss (i.e., those that showed the smallest step between the eroded and treated areas) were the novel Ca^2^-MSN and NaF groups. The control group presented the largest step and a visible degree of surface loss. The color scale of the 3D profilometry images confirmed that steps had formed between the eroded and treated areas in each product group (Figure 3).

## Discussion

The present study aims to compare the effects of a novel Ca^2^-MSN and other commonly used products on dental erosion. In present study, the novel Ca^2^-MSN exhibited a greater degree of effectiveness in reducing the progression of a previously installed erosion in a manner similar to the NaF treatment, as observed based on the TSL measurements of the eroded versus treated areas. Both the novel Ca^2^-MSN and NaF solutions correlated with a more minor loss of tooth structure than did the other products (i.e., CPP-ACP, CPP-ACP/F^−^, and TiF_4_) and water (negative control).

To our knowledge, this is the first study to incorporate calcium into a novel mesoporous silica nanoparticle applied to act as a limiting factor in the progression of dental erosion. The positive results obtained herein in regard to reducing the loss of tooth structure in comparison with other products may possibly be due to the fact that the porous structure of the silica encapsulates the to-be-released compounds, acting as a stabilizer and increasing the reaction by gradually releasing the compounds, thus creating chemical stability.^20,21^

Ca^2^-MSN showed results as positive as NaF, which is a product considered the gold standard in the use of remineralization. As a result, Ca^2^-MSN can be considered an important product to be incorporated into future *in vivo* studies, since it has benefits and positive results as seen in the present study. It is a product with high substantivity, containing many pores that allows a greater incorporation of several substances (in the case of our study calcium was chosen), to be a compound that has a synthesis using a simple process (providing a good cost /benefit), to be a biocompatible product in humans and in addition ,for having a great benefit for its chemical stability capacity, ^22,23^ keeping its properties stable even in the oral cavity that undergoes changes in temperature and pH.

When analyzing the fluoride compounds, positive results were obtained regarding the use of NaF, which can possibly be explained by the fact that fluoride decreases the solubilization of dental enamel (hydroxyapatite) through the formation of CaF_2_, leading to establishment of a barrier that is the first to be diluted after an erosive challenge. However, in the present study, TiF_4_ was not as effective as NaF in reducing the progression of enamel erosion. Nevertheless, we can speculate that TiF_4_ may still be able to prevent dental erosion.^24,25^As previously shown by Chevitarese, et al.^26^(2004), titanium penetrates more easily into the surface of sound enamel, i.e., into more regular surfaces. Of note, it is possible that the Tif_4_ layer may have been thinner or not continuous in our study in areas where a previously eroded surface already existed. A previous study about erosion prevention, found better results for TIF_4_ that was indeed able to act positively in preventing erosion^27^ than in the present study, which evaluated the minimization of the progression of dental erosion. Another reason why the results weren’t positive may have been that TiF_4_ has an acidic composition, with low pH values (pH: 1–2), which further damaged the previously eroded surface.^28^

In the present study, calcium-based products such as CPP-ACP and CPP-ACP/F− were used in comparison with Ca^2^-MSN. Both CPP-ACP products were not as effective as Ca^2^-MSN in protecting already eroded tooth enamel; when related to caries these products usually have a more beneficial effect than when used against dental erosion. Positive results are usually shown in many studies using CPP-ACP and CPP-ACP/F- in reducing the progression of caries lesions and increasing resistance to demineralization.^29,30^ According to Reynolds^31^(2009) and Rose^32^(2000), this is due the compound CPP-ACP includes a technology that adheres to the plaque, providing a reserve of calcium and phosphate. However, no benefits were identified as existing for either CPP-ACP and CPP-ACP/F^−^ regarding preventing dental plaque by creating a reservoir of phosphate and calcium ions in the present study, and neither compound presented a similar efficacy against tooth erosion as compared with Ca^2^-MSN and NaF. In addition, the flow addition product had a concentration of 900 ppm of F-, which may have been the differential factor, since the CPP-ACP/F- showed more erosion, compared to the positive controls that had 6,153- and 6,135-ppm concentrations of F-, respectively.

In addition, the lesser effectiveness in our study, of CPP-ACP and CPP-ACP/F− products, when compared to the use of more used products such as NaF for example, may have been due to the chosen time of application, studies that chose application times that varied between 3 to 5 minutes, showed better results when compared to our study, for example.^33-35^

## Conclusions

Based on the results of the present study, the discussed novel Ca^2^-MSN treatment can prevent the progression of enamel erosion as well as NaF can, likely due to the capacity of the silica to increase the bioavailability and slow the release of the incorporated molecules or ions, ^27^ much like the calcium used in our study, thus acting in the remineralization process. These positive results reveal an array of new possibilities to be tested in future *in vitro, in situ* and *in vivo* studies in order confirm the promising viability of Ca^2^-MSN.
